# Automatic, wearable-based, in-field eating detection approaches for public health research: a scoping review

**DOI:** 10.1038/s41746-020-0246-2

**Published:** 2020-03-13

**Authors:** Brooke M. Bell, Ridwan Alam, Nabil Alshurafa, Edison Thomaz, Abu S. Mondol, Kayla de la Haye, John A. Stankovic, John Lach, Donna Spruijt-Metz

**Affiliations:** 1 Department of Preventive Medicine, Keck School of Medicine, University of Southern California, Los Angeles, CA 90089 USA; 2 Department of Electrical and Computer Engineering, School of Engineering and Applied Science, University of Virginia, Charlottesville, VA 22904 USA; 3 Department of Preventive Medicine, Feinberg School of Medicine, Northwestern University, Chicago, IL 60611 USA; 4 Department of Computer Science, McCormick School of Engineering, Northwestern University, Chicago, IL 60611 USA; 5 Department of Electrical and Computer Engineering, Cockrell School of Engineering, The University of Texas at Austin, Austin, TX 78712 USA; 6 Department of Computer Science, School of Engineering and Applied Science, University of Virginia, Charlottesville, VA 22904 USA; 7 Department of Electrical and Computer Engineering, School of Engineering and Applied Science, The George Washington University, Washington, DC 20052 USA; 8 Center for Economic and Social Research, Dornsife College of Letters, Arts, and Sciences, University of Southern California, Los Angeles, CA 90089 USA; 9 Department of Psychology, Dornsife College of Letters, Arts, and Sciences, University of Southern California, Los Angeles, CA 90089 USA

**Keywords:** Translational research, Obesity

## Abstract

Dietary intake, eating behaviors, and context are important in chronic disease development, yet our ability to accurately assess these in research settings can be limited by biased traditional self-reporting tools. Objective measurement tools, specifically, wearable sensors, present the opportunity to minimize the major limitations of self-reported eating measures by generating supplementary sensor data that can improve the validity of self-report data in naturalistic settings. This scoping review summarizes the current use of wearable devices/sensors that automatically detect eating-related activity in naturalistic research settings. Five databases were searched in December 2019, and 618 records were retrieved from the literature search. This scoping review included *N* = 40 studies (from 33 articles) that reported on one or more wearable sensors used to automatically detect eating activity in the field. The majority of studies (*N* = 26, 65%) used multi-sensor systems (incorporating > 1 wearable sensors), and accelerometers were the most commonly utilized sensor (*N* = 25, 62.5%). All studies (*N* = 40, 100.0%) used either self-report or objective ground-truth methods to validate the inferred eating activity detected by the sensor(s). The most frequently reported evaluation metrics were Accuracy (*N* = 12) and F1-score (*N* = 10). This scoping review highlights the current state of wearable sensors’ ability to improve upon traditional eating assessment methods by passively detecting eating activity in naturalistic settings, over long periods of time, and with minimal user interaction. A key challenge in this field, wide variation in eating outcome measures and evaluation metrics, demonstrates the need for the development of a standardized form of comparability among sensors/multi-sensor systems and multidisciplinary collaboration.

## Introduction

Dietary intake (i.e., what and how much is consumed), eating behaviors (i.e., food choices and motives, feeding practices), and context (i.e., who is eating, when, where, with whom, etc.) play a significant role in the development of chronic diseases, including type 2 diabetes, heart disease, and obesity^1–6^. Recent data from the National Health and Nutrition Examination Survey (NHANES) indicate that national obesity prevalence for U.S. adults (39.6%) and for U.S. youth (18.5%) is the highest ever documented (compared with 14.5% and 5.0%, respectively, in the early 1970s)^7–9^. Poor diet is estimated to have contributed to 11 million deaths globally in 2017^10^. Despite this strong association between food intake and health, our ability to accurately assess dietary intake, eating behaviors, and context are three existing challenges in dietary research. Dietary intake/eating behavior assessment in both youth and adult populations historically relies on self-reporting tools^11,12^. The most commonly used tools to assess dietary intake/eating behaviors are 24-h recalls, food records (food diaries), and food frequency questionnaires (FFQ)^13,14^. Major limitations of these methods include participant burden and recall or memory bias^15,16^, which can lead to under- and over-reporting of dietary intake, skewing research findings in both directions^17^. Measurement tools that can minimize these limitations are crucial for accurately detecting temporal patterns of food and nutrient intake and having measurement sensitivity to detect intake changes, and to ultimately discern the influences of dietary intake and eating behaviors on health outcomes.

Traditional methods of eating assessment typically summarize dietary measures at the hour-, day-, week-, or even year-level^11,14^. Although these can be helpful in understanding relationships between eating behavior and its predictors, important micro-level temporal patterns and processes are not measured nor can they be explored with these measures. The ability to explore micro-level eating activities, such as meal microstructure (the dynamic process of eating, including meal duration, changes in eating rate, chewing frequency, etc.)^18^, food choices^19^, and processes (e.g., eating rate^20^; eating mimicry^21,22^; etc.), is important because recent literature suggests that they may play an important role on food selection, dietary intake, and ultimately, obesity and disease risk.

Emerging technologies present the opportunity to improve these assessment methods by using methods that improve the quality and validity of data that is collected, and by passively measuring eating activity in naturalistic settings over long periods of time with minimal user interaction. Technological advances in dietary assessment tools include: (i) web-based self-administered 24-h recall tool, which aims to reduce respondent burden^23^; (ii) mobile device-assisted ecological momentary assessment (mEMA), which focuses on reducing recall bias by collecting real-time data in naturalistic settings^24^; (iii) photo-assisted and image-based dietary assessments, which attempt to reduce respondent burden and recall bias^25^; and (iv) wearable sensors, which offer a suite of measurement tools that seek to tackle all of these limitations^26,27^. Wearable devices with embedded sensors in particular allow for the passive collection of various data streams that can be used to develop algorithms to infer eating behaviors in naturalistic settings and, with some types of sensors, over long periods of time. Collecting near-continuous data in the context of daily life, where behavior actually occurs, has proven to be extremely difficult for researchers and burdensome for participants^26,27^. However, wearable sensors can lessen the burden by passively collecting data while users go about their daily lives with minimal user input, compared with existing methods.

In the past decade, a range of wearable sensors for the purpose of automating eating detection have been proposed and studied. However, these studies have been primarily conducted in a combination of controlled lab and semi-controlled field settings^28,29^, and for good reason: these systems are challenging to develop, deploy, and evaluate. More recently, however, the research field has rapidly expanded the testing of these devices in the field. Previous research has shown significant differences in eating metrics (e.g., duration of meals, number of bites, etc.) between similar in-lab and in-field studies^30^, illustrating the importance of in-field testing and in-field validation of wearable sensors. Field deployment is crucial because it is where humans are more likely to behave naturally as compared with a research lab setting, and many of the influences on eating behavior cannot be replicated in a laboratory. Moreover, non-eating behavior that confounds these sensors (e.g., smoking, biting nails, etc.) are too many, and not all known, to replicate in a natural way in controlled settings. The data from wearable sensors deployed in the field will offer researchers a wealth of temporally-rich, highly contextualized eating activity data that can address exciting and novel research questions that were previously inexplorable.

The next step toward integrating these wearable technologies into public health research is to continue testing the wearable technology in the field, while beginning to address and solve the unique technical, analytical, and multidisciplinary challenges that arise from this effort. Wearable sensors provide the opportunity to examine and understand real-time eating behavior in context, but their introduction into field testing has been slow and challenging. It is unclear what kind of information is available in the literature about the unique features and challenges of field testing. Moreover, the measures with which these sensors are reported and evaluated is also highly varied and non-uniform. Therefore, a scoping review was conducted in order to:

1. catalog the current use of wearable devices and sensors that automatically detect eating activity (dietary intake and/or eating behavior) specifically in free-living research settings;
2. and identify the sample size, sensor types, ground-truth measures, eating outcomes, and evaluation metrics used to evaluate these sensors.

It was outside the scope of this paper to systematically review contextual factors, if any, detected with these automatic, wearable-based methods; however, a brief discussion on the potential for these methods in future research is included. In addition, a discussion of key challenges of in-field testing and recommendations for future research directions are included.

A scoping review was chosen due to the heterogenous methods used in this field of research.

## Methods

The reporting of this scoping review adheres to the Preferred Reporting Items for Systematic reviews and Meta-Analyses Extension for Scoping Reviews (PRISMA-ScR) checklist^31^.

### Information sources

We conducted a literature search of the PubMed, SCOPUS, IEEE Xplore, ACM Digital Library, and Google Scholar (first 50 results) databases for all literature published through December 2019. A research librarian assisted with the literature search strategy, which was further refined by the authorship team. The records retrieved from the databases were imported into a web-based systematic review management tool, Covidence^32^. We also hand searched the reference lists of the originally included publications for additional eligible studies.

### Literature search

The literature search strategy included a combination of keywords and variations to identify articles that addressed (i) eating assessment/detection, (ii) wearable technology, and (iii) free-living research settings. Keywords included “eat”, “food intake”, “diet”; “monitor”, “assess”, “detect”; and “wearable”, “device”, “sensor”, “technology”, “smart watch”, “smartwatch”, “ambulatory”, “free living”, “in field”, “in the wild”. The full search term strategy that was used for each database is outlined in Supplementary Table 1.

### Eligibility criteria

Peer-reviewed journal or conference papers were considered for inclusion in the review if they were published prior to December 22, 2019 and were written in English. Furthermore, eligible papers needed to have described/reported on all of the following components within the contents of the paper:

1. Any wearable device or sensor (i.e., worn on the body) that was used to automatically (i.e., no required actions by the user) detect any form of eating (e.g., content of food consumed, quantity of food consumed, eating event, etc.). Proxies for “eating” measures, such as glucose levels or energy expenditure, were not included. A “device” is defined as a wearable with embedded sensors that senses the body or environment and records data either on-device or externally through Bluetooth.
2. “In-field” (non-lab) testing of the sensor(s), in which eating and activities were performed at-will with no restrictions (i.e., what, where, with whom, when, and how the user ate could not be restricted). There was no limitation on the length of in-field testing. Furthermore, studies in which participants completed lab procedures (e.g., sensor calibration) followed by an in-field observation period that included eating were included. Studies that were described as semi-free living (e.g., participants wore device in the wild without restriction, but ate their meals in the lab), or studies that did not fit our aforementioned definition of “in-field”, were excluded.
3. At least one evaluation metric (e.g., Accuracy, Sensitivity, Precision, F1-score) that indicated the performance of the sensor on detecting its respective form of eating.

Review articles, commentary articles, study protocol articles, and any other articles without reported results from empirical research were excluded.

### Screening and selection of articles

The records retrieved from the databases were imported into a web-based systematic review management tool, Covidence^32^. Any duplicate articles were removed.

**Fig. 1:**
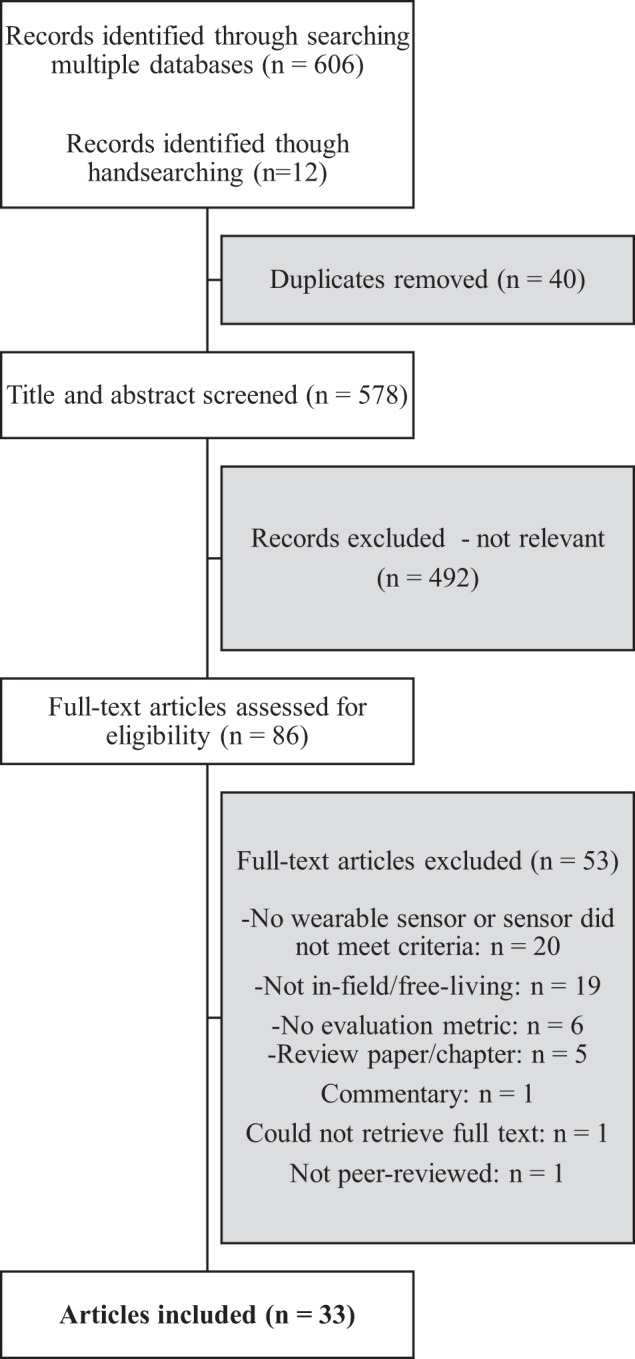
Flow diagram of article selection process.

First, the titles and abstracts of all articles were reviewed. Articles were excluded at this initial screening phase if they did not describe at least one wearable device or sensor that was used to automatically detect any form of eating. If this information could not be ascertained from the title and/or abstract, the article’s full text was reviewed in the next screening phase to determine whether it fit the eligibility criteria. Furthermore, if the abstract indicated that the study was not conducted in the field under free-living conditions, it was excluded. Otherwise, this criterion was assessed in the full-text screening phase (Fig. 1).

After the initial title/abstract screening process, the full texts of any remaining articles were assessed. Articles were further excluded if they did not meet the remaining eligibility criteria, including studies being conducted in free-living conditions; reporting at least one evaluation metric of the sensor’s performance; being peer-reviewed; and reporting results from empirical research (Fig. 1).

### Data extraction and synthesis

If any research article reported on more than one in-field study that fit our eligibility criteria (and contained different subject samples), then all eligible studies from that article were included in our review as separate studies.

Two research assistants independently extracted the following study characteristics from the final set of eligible studies using a custom-made data extraction worksheet. The extracted study characteristics were reviewed by first author BMB, and discrepancies were discussed and resolved by authors BMB and RA.

1. Study sample size: the total number of participants for each study.
2. Length of data collection period in free-living environment: how long the participants wore the sensor(s) (i.e., participated in the study) in the field.
3. Number of wearable sensors used to detect eating: the total number of wearable sensors in which the sensor’s signal data were used, at least in part, to detect eating. Any other equipment that was part of the overall sensor system, but did not ultimately contribute signal data to detect eating, was not included in this count.
4. Types of wearable sensor(s) used to automatically detect eating: the type of sensors embedded within the selected wearable devices in which the sensor’s signal data were used, at least in part, to detect eating.
5. Ground-truth method(s): the method, if any, that was used in the study to evaluate the performance of the sensor(s) in the free-living environment.
6. Form of eating activity (eating outcome) measured by the sensor(s): eating outcomes that were at least partially derived from sensor signal data and had a corresponding evaluation metric. In some cases, multiple eating outcomes for a single study were included. Terminology used to describe the eating outcome in the original article was maintained in our reporting.
7. Evaluation metric(s) of the sensor(s): any evaluation metric, reported either in text, a table, or a figure, that described the performance of the wearable sensor(s) on detecting eating in free-living settings. If the exact numerical value was not reported or could not be ascertained from a figure, and it was not reported numerically anywhere else in the text, then this evaluation metric was not included in Table 1. Only evaluation metrics that were exclusively for eating activity detection were included. Evaluation metrics that included the detection of other activities (e.g., sleeping, exercising, etc.) were not included. Often, multiple metrics were reported for different methods/algorithms deployed. If the article’s authors indicated or highlighted the best performing method or algorithm result, then this was selected and included in the table for each unique method. Otherwise, the best performing (typically the highest value) of each unique method was identified in the article by the first author (BMB) and included in Table 1. Last, we exclusively included the metrics reported in the studies, and did not calculate any metrics, even if calculations were possible. For example, if Sensitivity and Specificity were reported, but Accuracy was not reported, we did not calculate Accuracy even though possible with the reported data.

**Table 1:** Methods and performance reported in wearable-based eating detection research studies (*N* = 40).

First author, Year	Sample size	Free-living session duration	No. of sensor types	Sensor type(s)	Ground-truth method(s)	Eating outcome(s)	Select evaluation metric(s)^a^
Bedri, 2015^33^	6	6 h	2	(1) Gyroscope (2) Infrared (IR) proximity sensor	Activity log	Eating events	Accuracy = 82.3% Avg. false positives/h: 1.7 Precision = 41.7% Recall = 90.1%
Bedri, 2017^34^	10	3 h (×2 days)	3	(1) Inertial measurement unit (IMU)^b^ (2) IR proximity sensor (3) Microphone	Wearable video camera (1-s resolution)	Chewing	Accuracy = 93% F1-score = 80.1% Precision = 81.2% Recall = 79%
						Eating episodes	False positives: 2 True Positives: 15 (of 16)
Bi, 2018^35^	14	2 h	1	(1) Microphone	Wearable video camera (1-s resolution)	Eating episodes	*Jaccard similarity coefficient:* Correctly detected episodes: 20 (of 26) Falsely detected episodes: 12 Missed episodes: 6 (of 26) *Ward’s metrics:* Correctly detected episodes: 24 (of 26) Deletions: 2 (of 26) False insertions: 12
Blechert, 2017^36^	14	1 day and 1 night	1	(1) Electromyogram (EMG) electrodes	Eating log via smartphone app; marker button on recording device	Eating episodes	Sensitivity = 87.3% Specificity = 86.9%
Chun, 2018^37^	15	1 day	2	(1) Accelerometer (in smartphone) (2) IR proximity sensor	Eating log via smartphone app	Eating episodes	Precision = 78.2% Recall = 72.5%
Dong, 2011^38^	4	1 day	3	(1) Accelerometer (2) Gyroscope (3) Magnetometer	Activity log	Eating activity	False negatives: 3 False positives: 6 Positive predictive value = 70% Sensitivity = 82%
Dong, 2014^39^	43	1 day	2	(1) Accelerometer (in smartphone) (2) Gyroscope (in smartphone)	Eating log (first 20 participants); eating log via smartphone marker button (remaining 23 participants)	Meals/snacks	Accuracy = 81% Sensitivity = 81% Specificity = 82%
Doulah, 2017^18^	8	24 h	3	(1) Accelerometer (2) Piezoelectric strain gauge sensor^c^ (3) Radio-frequency (RF) transmitter and receiver	Eating log; push button	Eating episode duration	*Bland-Altman analysis:* Good agreement between automatic ingestion monitor & push button, but poor agreement between eating log and other methods. *Multiple comparisons analysis:* Eating episode durations from eating log were sig. diff. from automatic ingestion monitor (*p* < 0.001) and push button (*p* < 0.001).
Farooq, 2013^40^	12	24 h	3	(1) Accelerometer (2) Piezoelectric strain gauge sensor^c^ (3) RF transmitter and receiver	Activity log; push button	Food intake	*Artificial Neural Network technique:* Accuracy = 86.86% Precision = 87.59% Sensitivity = 86.13% *Support Vector Machine technique:* Accuracy = 81.93% Precision = 83.76% Sensitivity = 80.10%
	1	~48 h	3	(1) Accelerometer (2) Piezoelectric strain gauge sensor^c^ (3) RF transmitter and receiver	Activity log	Meal episodes	*Artificial Neural Network technique:* Correctly identified episodes: 8 (of 11) False positives: 1 *Support Vector Machine technique:* Correctly identified episodes: 7 (of 11) False positives: 3
Farooq, 2016^41^	12	24 h	3	(1) Accelerometer (2) Piezoelectric film sensor^c^ (3) RF transmitter and receiver	Activity log; push button	Food intake	*Fisher’s Linear Discriminate Analysis (Bagging technique):* Accuracy = 93.11% Precision = 96.72% Recall = 89.51%
Farooq, 2017^42^	8	≤3 h	1	(1) Piezoelectric film sensor	Portable tally counter; push button	Chews	Chew count estimation error (mean absolute value) = 6.24%
Farooq, 2018^43^	8	≤3 h	1	(1) Accelerometer	Eating log; push button	Food intake	F1-score = 85.8% Precision = 88.6% Recall = 85.4%
Fontana, 2013^44^	12	24 h	3	(1) Piezoelectric strain gauge sensor^c^ (2) RF transmitter and receiver	Push button	Food intake	Accuracy = 73.2%
Fontana, 2014^45^	12	24 h	3	(1) Accelerometer (2) Piezoelectric film sensor (3) RF transmitter and receiver	Eating log; push button	Food intake	*Using piezoelectric sensor and accelerometer data only:* Accuracy = 89.8% Precision = 89.8% Recall = 89.9%
Fortuna, 2016^46^	3	Several hours	2	(1) Accelerometer (2) Gyroscope	Wearable video camera (10-s resolution)	Hand-to-mouth motions (“bites”)	*Subjects 1 and 2:* Accuracy = 96.9% Precision = ~70% Recall = ~70% *Subject 3:* Accuracy = ~82% Precision = ~18% Recall = ~70%
Gao, 2016^47^	4	More than 10 days	1	(1) Microphone (in Bluetooth headset)	Participants recorded their eating episodes with a smartphone front-facing video camera	Eating episodes	*Leave One Sample Out (LOSO) method:* Accuracy = 75.61% Deep learning accuracy = 94.72% *Leave One Person Out (LOPO) method:* Accuracy = 65.43% Deep learning accuracy = 76.82%
Gomes, 2019^48^	5	3–5 h	2	(1) Accelerometer (in IMU) (2) Gyroscope (in IMU)	Real-time annotation via mobile phone prompt	Hand-to-mouth movements preceding a drinking event	False negatives = 24 False positives = 27 True positives = 113 F-score = 0.85 Precision = 0.84 Recall = 0.85
Hamatani, 2018^49^	16	1 day	2	(1) Accelerometer (in smartwatch) (2) Gyroscope (in smartwatch)	Wearable video camera (5-s resolution)	Drinking activity	False negatives: 40 False positives: 31 True positives: 138 Precision = 81.7% Recall = 77.5%
						Fluid intake	Mean absolute percentage error = 31.8% Mean percentage error = 14.0% Overall error = 4.3%
	8	2 days	2	(1) Accelerometer (in smartwatch) (2) Gyroscope (in smartwatch)	Wearable video camera (5-s resolution)	Drinking activity	False negatives: 33 False positives: 41 True positives: 81 Precision = 66.4% Recall = 71.1%
						Fluid intake	Mean absolute percentage error = 34.6% Mean percentage error = 6.9% Overall error = −15.9%
Jia, 2018^50^	1	1 week	1	(1) Wearable video camera	Wearable video camera (10-s resolution)	Food and drink images (episodes)	*When Burden Index* = *18% (k* = *2):* Sensitivity = 74.0% Specificity = 87.0%
Kyritsis, 2019^51^	6	Not reported (on average, 17,398 s per person)	2	(1) Accelerometer (in smartwatch) (2) Gyroscope (in smartwatch)	Eating log	Meals	False negatives = 55,083 (s) False positives = 47,187 (s) True negatives = 6,424,247 (s) True positives = 432,917 (s) Average Jaccard Index = 0.804 F1-score = 0.894 Precision = 0.901 Recall = 0.887 Specificity = 0.992
Mirtchouk, 2017^52^	5	12 h (×2 days)	3	(1) Inertial motion sensor (in smartwatch) (2) Microphone (3) Motion sensor (in Google Glass)	Eating log; photos of meals; voice notes	Meals	Accuracy = 85% Precision = 31% Recall = 87%
	6	2 or 5 days	2	(1) Inertial motion sensor (in smartwatch) (2) Microphone	Eating log; photos of meals; voice notes	Meals	Accuracy = 79% Precision = 25% Recall = 83%
Navarathna, 2018^53^	1	1 month	4	(1) Accelerometer (in smartphone) (2) Accelerometer (in smartwatch) (3) GPS (in smartphone) (4) Gyroscope (in smartwatch)	Activity log via smartphone app	Eating activity	*Using gyroscope data only:* Correctly predicted eating activity: 1206
Rahman, 2016^54^	8	5 days	4	(1) Affectiva Q Sensor^d^ (2) GPS (in smartphone) (3) Microphone (4) Microsoft Band^e^	Eating log via smartphone app	“About-to-Eat” moments	F-score = 0.69 Precision = 0.67 Recall = 0.77
Schiboni, 2018^55^	1	4 days	1	(1) Wearable video camera	Wearable video camera (1-s resolution)	Dietary events	F1-score = 90% Mean average precision (mAP) = 51% Precision = 78% Recall = 100%
Sen, 2017^56^	7	5 days	3	(1) Accelerometer (in smartwatch) (2) Camera (in smartwatch) (3) Gyroscope (in smartwatch)	Smartwatch camera (participants validated the images via smartphone food journal at end of the day)	Eating periods	False negatives = 0% False positives = 60.3% True positives: 31
	6	2 days	3	(1) Accelerometer (in smartwatch) (2) Camera (in smartwatch) (3) Gyroscope (in smartwatch)	Smartwatch camera (participants validated the images via smartphone food journal at end of the day)	Eating periods	False negatives = 35.3% False positives = 31.3% True positives: 11
	4	5 days	3	(1) Accelerometer (in smartwatch) (2) Camera (in smartwatch) (3) Gyroscope (in smartwatch)	Smartwatch camera (participants validated the images via smartphone food journal at end of the day)	Eating periods	False negatives = 3.3% False positives = 23.7% True positives: 29
Sen, 2018^57^	4	5 days	3	(1) Accelerometer (in smartwatch) (2) Camera (in smartwatch) (3) Gyroscope (in smartwatch)	Smartwatch camera (participants validated the images via smartphone food journal at end of the day)	Eating episodes	False negatives: 1 (of 30) False positives: 2 True positives: 29 (of 30)
	5	4–6 days	3	(1) Accelerometer (in smartwatch) (2) Camera (in smartwatch) (3) Gyroscope (in smartwatch)	Smartwatch camera (participants validated the images via smartphone food journal at end of the day)	Meals	False negatives: 3 (of 51) False positives: 2 True positives: 48 (of 51) *Combined (study 1* + *2):* Precision = 95% (after image filtering) Recall = 95%
Sharma, 2016^58^	104	1 day	2	(1) Accelerometer (2) Gyroscope	Eating log	Eating activity	Accuracy = 75% Sensitivity = 69% Specificity = 80%
Thomaz, 2015a^59^	7	Not reported (on average, 5 h 42 minutes per person)	1	(1) Accelerometer (in smartwatch)	Wearable video camera (60-s resolution)	Eating moments	F-score = 76.1% Precision = 66.7% Recall = 88.8%
	1	31 days	1	(1) Accelerometer (in smartwatch)	Wearable video camera (60-s resolution)	Eating moments	F-score = 71.3% Precision = 65.2% Recall = 78.6%
Thomaz, 2015b^60^	20	4–7 h	1	(1) Microphone	Activity log	Meal eating activity	F-score = 79.8%; Precision = 89.6%; Recall = 76.3%;
Yatani, 2012^61^	5	1 day	1	(1) Microphone	Wearable video camera (in smartphone) (resolution not reported)	Eating activity	*Support Vector Machine classification:* Correctly predicted eating activities: 157 Precision = 81.3% Recall = 87.8% *Naive Bayes classification:* Correctly predicted eating activities: 125 Precision = 62.2% Recall = 69.8%
						Drinking activity	*Support Vector Machine classification:* Correctly predicted drinking activities: 33 Precision = 61.1% Recall = 56.0% *Naive Bayes classification:* Correctly predicted drinking activities: 36 Precision = 28.6% Recall = 61.0%
Ye, 2016^62^	7	2 weeks	1	(1) Accelerometer (in smartwatch)	Eating log in Evernote app; smartwatch physical button pushed to confirm/deny eating activity	Eating	False detections per subject per day: ~7 Precision = ~31%
Zhang, 2018a^63^	10	1 day	1	(1) EMG electrodes	Eating log	Eating events	F1-score = 95.2% Precision = 98.2% Recall = 98.7%
Zhang, 2018b^64^	10	1 day	1	(1) EMG electrodes	Activity log	Chewing	Precision = 77.3% Recall = 78.8%
						Eating events	False negatives: 1 (of 44) False positives: 0 True positives: 43 (of 44) Precision > 0.95 Recall > 0.95

^a^The highest reported value for each unique evaluation metric is reported in this table. If multiple methods and/or algorithms were evaluated within a single study, the highest reported value for each unique evaluation metric for each method is reported.

^b^An inertial measurement unit (IMU) is a device that is typically comprised of a combination of accelerometers, gyroscopes, and sometimes magnetometers.

^c^Additional information needed to adequately describe this sensor was extracted from a paper previously published by the same author, in which the sensor is described in more detail (Sazonov and Fontana^83^).

^d^The Affectiva Q sensor measures electrodermal activity.

^e^The Microsoft Band is a smartwatch/fitness tracker that contains many sensors, including an accelerometer, gyroscope, heart rate monitor, and skin temperature sensor.

### Methods of summarizing data

Mean and standard deviation were calculated for each extracted continuous variable (sample size, length of time for data collection, number of sensor types, evaluation metric). Frequency tables were constructed for each extracted categorical variable (sensor types, ground-truth methods, eating outcomes).

Furthermore, the reported eating outcomes were grouped into three categories:

i. inferred eating occasions: the incident or event of eating activity;
ii. inferred chews (mastication): the grinding of food into smaller pieces by teeth;
iii. inferred hand-to-mouth gestures: the placement of food into the mouth via movement of the hand to the mouth (also referred to as “bite”).

These three categories are not exhaustive of all possible types of eating outcome categories, but rather only represent those that were derived from this review’s included studies.

## Results

### Literature search

The literature search produced 618 research articles, with 40 duplicates, resulting in 578 articles to be screened. After reviewing all article titles and abstracts, the full texts of 86 of these articles were further reviewed for eligibility. After removing articles that did not meet the inclusion criteria (see Fig. 1 for full list of exclusion reasons), 33 articles were deemed eligible for the review^18,33–64^ (Fig. 1). Six of these articles^40,49,52,56,57,59^ reported on more than one in-field study that fit our eligibility criteria, so *N* = 40 studies (from 33 articles) is considered to be the final sample size for the review.

### Study characteristics

The earliest publication year of the reviewed papers was 2011, and the most recent year was 2019. The sample size of the studies ranged from 1 to 104 participants, with a mean of 10.83 participants (SD = 16.73) per study (Table 1). The length of time for data collection in the “wild” environment varied and was not always reported with an exact numerical value or unit. Therefore, we will just report the range: 2 h to 1 month. The reported length of time for each study is available in Table 1.

### Wearable sensors

The majority of studies (*N* = 26 of 40) used multi-sensor systems (incorporating > 1 wearable sensor) to automatically detect eating activity^18,33,34,37–41,44–46,48,49,51–54,56–58^. On average, 2.10 wearable sensors (SD = 0.96) were used in the studies, with a range of 1–4 sensors (Table 1). Approximately 63% (*N* = 25) of the 40 studies utilized an accelerometer (device that determines acceleration) either by itself (*N* = 4) or incorporated into a sensor system (*N* = 21) to detect eating activity^18,37–41,43,45,46,48,49,51,53,56–59,62^ (Table 2). The second most frequently utilized wearable sensor was a gyroscope (device that determines orientation) (*N* = 15)^33,38,39,46,48,49,51,53,56–58^, followed by a microphone (*N* = 8)^34,35,47,52,54,60,61^, a piezoelectric sensor (*N* = 7)^18,40–42,44,45^, a RF transmitter and receiver (*N* = 6)^18,40,41,44,45^, and a smartwatch camera (*N* = 5)^56,57^ (Table 2). EMG electrodes^36,63,64^, a motion sensor^52^, and an infrared proximity sensor^33,34,37^ were used in three studies each. Wearable video cameras^50,55^ and GPS^53,54^ were used in two studies. Last, the Affectiva Q sensor (used to measure electrodermal activity)^54^, an inertial measurement unit (IMU)^34^, a magnetometer^38^, and a Microsoft Band^54^ were used in one study each (Table 2).

**Table 2:** Frequency and percentage of sensor types in included studies, ordered by frequency.

Sensor type	Frequency	Percentage (of 40 studies)
Accelerometer	25	62.50%
Gyroscope	15	37.50%
Microphone	8	20.00%
Piezoelectric sensor	7	17.50%
Radio-frequency transmitter and receiver	6	15.00%
Smartwatch camera	5	12.50%
Electromyogram electrodes	3	7.50%
Motion sensor	3	7.50%
Infrared proximity sensor	3	7.50%
GPS	2	5.00%
Wearable video camera	2	5.00%
Affectiva Q Sensor	1	2.50%
Inertial measurement unit	1	2.50%
Magnetometer	1	2.50%
Microsoft Band	1	2.50%

### Ground-truth methods

To evaluate the performance of the wearable sensors in automatically detecting eating in the field, 100% of studies (*N* = 40) used one or more validation methods in which the “ground-truth” eating data reported by the participants (self-report methods) or produced by other wearable devices (objective methods) were compared with the inferred eating activity detected by the sensor(s).

#### Self-report methods

Six of the studies had participants self-report all daily activities, including eating, via a log or diary^33,38,40,53,60,64^, while six studies had participants self-report just eating activity^37,39,51,54,58,63^. In one study, participants were asked to record their eating episodes with a smartphone front-facing video camera to obtain ground-truth eating^47^. In another study, participants used a push button, located on a wireless device, as the primary method for self-reporting food intake; participants pressed and held a button during chewing to indicate the start and end of a chewing bout^44^. In Farooq and Sazonov^42^, participants used a push button, and they also counted their number of chews during each chewing bout by using a portable tally counter (a device used to incrementally count something).

Other studies used multiple self-report methods to collect ground-truth eating activity. Six studies used both an eating or activity log in addition to a marker/push button^18,36,40,41,43,45^. Two studies, both reported in Mirtchouk et al.^52^, had participants use a combination of eating logs, voice notes (“spoken annotations”), and self-taken photos at the start and end of meals.

#### Objective methods

One-quarter of studies (*N* = 10 of 40)^34,35,46,49,50,55,59,61^ outfitted participants with a wearable video camera for the length of their respective data collection period, in which the video clips were later annotated by a person or persons from the research team to indicate periods of eating activity. The video cameras captured images at varying levels of resolution: three studies captured images in 1-s intervals; two studies captured images every 5 s; two studies captured images every 10 s; and two studies captured images every 60 s.

#### Mixed methods

Two studies sent participants real-time messages to confirm whether or not they were eating. In Ye et al.^62^, participants were sent a short message on their smartwatch if an eating gesture was detected; participants were able to confirm or reject if they were eating in real-time. Similarly, in Gomes and Sousa^48^, when drinking activity was detected, participants were sent an alert on their smartphone and could then confirm or reject if they were drinking in real-time.

Last, five studies (reported in two papers) used an “automated food journaling system”, which consisted of a combination of self-report (food journal), wearable sensor (smartwatch inertial sensors), and objective data (smartwatch camera) to discern ground-truth of eating activity in real-time^56,57^. When the wrist-worn smartwatch detected an eating episode, the smartwatch then captured images and sent them to a server. After image processing techniques were applied to eliminate irrelevant images, a subset of relevant images were stored in the server and could be viewed by the user to assist with their self-report online food journaling^56,57^.

### Eating outcomes

Authors collectively reported on 22 different types of eating outcomes (Table 1). For each study, only the eating outcome that was (a) directly measured by the wearable sensors, and (b) evaluated with comparison to a ground-truth method is included in Tables 1 and 3 and is reported in this section. Five studies^34,49,61,64^ reported two eating outcomes each that fit these criteria, therefore the five additional outcomes were included, totaling 45 eating outcomes.

**Table 3:** Reported eating outcomes (*n* = 45) from included studies, by category.

Category 1: Eating occasions (*n* = 40)	Category 2: Chews (*n* = 3)	Category 3: Hand-to-mouth gestures (*n* = 2)
“About-to-Eat” moments: 1	Chewing: 2	Hand-to-mouth motions: 1
Dietary events: 1	Chews: 1	Hand-to-mouth movements preceding a drinking event: 1
Drinking activity: 3		
Eating: 1		
Eating activity: 4		
Eating episode duration: 1		
Eating episodes: 6		
Eating events: 3		
Eating moments: 2		
Eating periods: 3		
Fluid intake: 2		
Food & drink images (episodes): 1		
Food intake: 5		
Meal eating activity: 1		
Meal episodes: 1		
Meals: 4		
Meals/snacks: 1		

The reported measures were grouped into three categories: (1) inferred eating occasions, (2) inferred chews, and (3) inferred hand-to-mouth gestures (Table 3). The majority of studies (*N* = 37 of 40) used wearable sensors to infer eating occasions (Category 1), however, these measures were often referred to in different terms (e.g., “eating event” vs. “eating episode” vs. “dietary event”). Three studies inferred chews (Category 2)^34,42,64^ and two studies inferred hand-to-mouth gestures (Category 3)^46,48^.

### Sensor evaluation metrics

Studies often reported multiple and varied evaluation metrics. All reported evaluation metrics for their corresponding eating outcome(s) are included in Table 1. The most frequently reported metrics were Accuracy and F1-score. Twelve studies reported Accuracy^33,34,39–41,44–47,52,58^ and 10 studies reported F1-score^34,43,48,51,54,55,59,60,63^. Due to the lack of a standardized evaluation metric across studies, we do not summarize (calculate mean, standard deviation, etc.) the reported metrics. However, select evaluation metric values for each study are available in Table 1.

## Discussion

Detecting and monitoring human eating behavior in the real-world is a growing field and offers exciting and novel opportunities for future public health research. Previous literature reviews on automatic eating detection methods have often placed a focus on sensor modalities and technologies in any research setting^28^, on specific sensor modalities, such as wearable video cameras^65^, or on specific sensor locations, such as the upper limb^29^. No review to date has focused specifically on studies that use wearable devices to automatically detect eating activity in naturalistic settings. Furthermore, this review has been written by a multidisciplinary team, with a multidisciplinary audience in mind. Below we provide a discussion on trends in wearable sensors for eating detection, a summary of technical, analytical, and multidisciplinary challenges that occur when conducting in-the-wild research, and recommendations for future public health research directions.

### Summary of key findings

This scoping review included 40 studies that reported on the use of wearable sensors to automatically detect eating in the field published through December 2019. The majority of studies utilized accelerometers (*N* = 25 of 40) and/or gyroscopes (*N* = 15 of 40) to automatically detect eating activity via wearable sensors. Self-reported eating was the most frequently used method for collecting ground-truth eating activity data, whereas objective methods such as wearable video cameras were used in only one-quarter of studies (*N* = 10 of 40).

Variations of ‘inferred eating occasions’ (e.g., eating episode, eating event, eating period) were the most commonly reported eating measure (*N* = 37), followed by inferred chews (*N* = 3), and then inferred hand-to-mouth gestures (*N* = 2). The most commonly reported evaluation metrics were Accuracy (*N* = 12) and F1-score (*N* = 10).

In reviewing the methods and performance of wearable devices and sensors that automatically detect eating in naturalistic research settings, several trends and major challenges can be identified.

### Trends, challenges, and recommendations

#### Sensors

In this review, we observe that early research efforts attempted to find novelty and improvement by experimenting with new sensor types and/or sensor locations. The first paper to report the use of a single sensor to detect eating was in 2012^61^, but it did not become more prevalent until 2015^59,60^; it became much more popular by 2018, in which more than half of the 10 papers published that year only used a single sensor^35,43,50,55,63,64^. With time, these efforts have converged into a shorter list of sensor types and locations with emphasis on two major criteria: the sensors’ ability to capture the patterns of eating outcomes, and the real-world practicality, which includes user comfort, acceptability, and accessibility of continuously wearing these sensors in a real-world setting for a long duration of time. As seen in Tables 1 and 2, the majority of studies utilized accelerometers (*N* = 25) and/or gyroscopes (*N* = 15), typically embedded within a wrist-worn smartwatch or device. This observation highlights the emergence of smartwatches as a potentially practical and user-friendly modality for real-world eating behavior monitoring. In addition to the user advantages, smartwatch-based methods also have engineering advantages, such as facilitating compact and concise form factor, containing reasonable computational and power resources (e.g., low battery footprint), and offering wide possibilities for adoption with the rising Internet of Things (IOT)-cloud-edge-based ubiquitous computing era.

Another noted trend is the combination of multiple sensors’ information from different modalities placed on various on-body locations (for example, the piezoelectric strain gauge-infrared proximity sensor-accelerometer combination used in a few studies^18,40,41,44^). However, using a ‘multi-sensor system’ (i.e., multiple sensors) as opposed to a single sensor introduces a multitude of challenges, ranging from having to integrate different sampling rates and signal amplitudes to issues arising from data synchronization (i.e., aligning sensor signals in time across multiple devices due to differences in clock times) and reliability (i.e., ensuring a consistent sampling rate to maintain high quality data throughout the study). Despite these challenges, this multi-sensor approach offers high potential for real-time monitoring and tracking of human eating behavior. Multi-sensor fusion can provide further context-awareness, which can aid in modeling of behavior; and in optimization of power (e.g., a low power sensor can trigger a higher power sensor only when necessary) and computational complexities, which is vital for sensor-driven monitoring in real-world settings. Furthermore, it can provide new opportunities for simultaneously detecting eating behavior (e.g., when people are eating), dietary intake (e.g., what people are eating), and context (e.g., where people are eating, with whom), culminating in a much fuller understanding of individual eating patterns and dynamics.

Two papers that were retrieved in the original literature search, but were not ultimately included in the review because they did not report an evaluation metric of the automatic eating detection performance, have started to do this. Vaizman et al. and Gemming et al. report on using wearable sensors and mobile devices to automatically capture both eating and contexts in natural environments (free living)^66,67^. In Gemming et al.^66^, social and contexts such as eating location, external environment (indoor/outdoor), physical position, social interaction, and viewing media screens are collected. Similarly, Vaizman et al.^67^ collect contexts such as location (e.g., in class, in a meeting, at a restaurant), activities (e.g., running, drinking (alcohol), eating), and company (e.g., with friends). These recent works suggest that automatic eating and context detection methods offer opportunities to provide valuable, rich, real-time information with important obesity-prevention research implications.

#### Ground-truth methods

Our review indicates that there is still a strong reliance on self-report as the method of determining ground-truth eating activity in the field. Although using objective methods such as wearable video cameras^68^ to determine when eating activity has occurred can be very costly and time-intensive, maintaining eating and/or activity logs is a process that is very burdensome on the participant and ultimately relies on their memory on when food was eaten. In studies comparing food diaries to the doubly labeled water method, which uses doubly labeled water as a biomarker for energy intake and is considered the “gold-standard” method^69^ of dietary assessment, there is evidence that misreporting of dietary intake is common^17^. Moreover, these “gold-standard” methods have recently been shown to exhibit methodological biases^70^, particularly when individuals consume low-carbohydrate diets; and have only been used to evaluate self-reported dietary intake, not self-reported timing of eating activity. The accuracy of self-reported timing of eating activity via food diaries is unknown. The aforementioned limitations in current eating assessment methodology were often cited in the reviewed articles as the motivating factor for developing wearable technologies that can overcome these constraints. Therefore, the use of these self-report methods to validate the wearable sensors may need to be reconsidered in the future when evaluating the reported performance of these sensors.

The trend in ground-truth validation is increasingly in favor of using a wearable video camera because of the increased confidence established from visually confirming the activity or behavior being performed; 10 of the included studies used wearable video cameras as ground-truth methods^34,35,46,49,50,55,59,61^, with six of those studies taking place in the last three years (2017–2019). However, privacy and stigma challenges associated with wearable video cameras remain when studying participants in free-living populations, particularly if the objective is to capture authentic eating behavior^71^. Several device- (i.e., video camera lens orientation, location, look and feel) and data-specific factors (i.e., what is being collected by the video camera) introduces discomfort, thereby influencing an individual’s willingness to act naturally and results in wearers modifying their behavior or abandoning the device^71^. However, these devices may only need to be worn for a limited time, to aid in the process of validating and building machine learning-based models that detect eating in the real world.

Mobile device-assisted ecological momentary assessment (mEMA), repeated sampling of one’s behavior in real-time and in-context^24^, has been suggested as a promising tool for eating assessment^72^, and may also serve as a novel tool to evaluate the performance of wearable sensors in the field. In a recent review, Schembre and colleagues^73^ summarize the existing literature on mEMA methods for the measurement of dietary intake in research studies. They conclude that mobile ecological momentary dietary assessment methods may be interchangeable with existing methods, in addition to reducing participant burden, recall biases, and technological barriers while maximizing ecological validity^73^.

Two studies (Ye et al.^62^, Gomes and Sousa^48^) included in this review used a novel method for obtaining ground-truth eating activity in the wild similar to mEMA. When an eating/drinking gesture was detected via the wearable sensors, the watch or phone displayed a short message asking the user to confirm or reject that the user was eating/drinking. Similarly, in a study not included in this review, Spruijt-Metz et al.^74^ report on an integrated system of wearable sensors and smartphones that collects family eating behavior data in the wild, and propose using mEMA to collect ground-truth eating data^75^. mEMA and similar methods do have their own limitations, such as relying on self-reported eating activity and the potential for low compliance, yet may offer the ability to capture and validate ground-truth eating activity in naturalistic settings for longer periods of time as compared with other subjective and objective measures, thus potentially improving research scalability and participant acceptability.

Exploration and implementation of new methods for acquiring ground-truth eating activity in naturalistic settings, as well as continued testing of the emerging methods such as wearable video cameras and mEMA, is warranted in future research in order to improve the validity and reliability of eating detection via wearable sensors.

### Limitations of the scoping review

To our knowledge, this is one of the first review papers to date that catalogs and summarizes the current use of wearable sensors that automatically detect eating activity specifically in free-living or uncontrolled research settings. However, due to this strict inclusion criteria, we did not include papers that reported on sensors deployed in semi-controlled settings, which may have contributed to a more comprehensive review of the current sensors being used in this field of research. In addition, the lack of both a standardized eating outcome (see Table 3) and a standardized evaluation measure of said eating activity prevented us from comparing successes across studies. Moreover, we selected and reported the ‘best’ evaluation metric from studies in Table 1 (typically the highest value), thus potentially biasing the overall takeaway message of how accurate these sensors are at detecting eating in the wild. Last, some evaluation metric results for the included papers were exclusively reported in figures (not textually or numerically), and were omitted from this review due to the data extraction protocol.

### From research to practice

Recent technological advances have provided engineers with the capability to develop sensors and devices that can collect the data needed to objectively infer eating activity in a variety of novel ways, such as inferring bites via data from wrist-worn sensors^46^, sensing jaw movement to infer chewing activity^42^, and utilizing the signal obtained from EMG electrodes to infer when eating events occur^63^. This variety of sensors and abundance of inferable eating outcomes, however, contributes to the difficulty of comparing performance across sensors and evaluation methods. Some form of comparability among similar modalities will be necessary in order to integrate wearable sensors into public health research. This includes developing some standardized form of comparability, such as standardized terminology and standardized evaluation metrics, or at least the sharing of algorithms that are used to infer various eating behaviors. Importantly, identification of meaningful eating outcomes and the development of the algorithms to detect these outcomes would benefit from being generated with input from relevant collaborators such as computer scientists and engineers, health behaviorists, and nutritionists.

Researchers within the public health field are increasingly interested in utilizing wearable sensors to assess eating activity. However, diet, which is one of the most important factors that contributes to overweight and obesity status and other diseases, is notoriously hard to assess with our current methods^16^. Although flawed, more traditional methods offer assurance in what type of eating outcome is being measured. For instance, 24-h dietary recalls typically obtain detailed information on all foods and beverages consumed on a given day, which can later be processed into meaningful behaviors (at least, meaningful as determined by the nutrition field) such as average total intake of kilocalories per day or an overall healthy eating index. As indicated by the numerous eating outcomes extracted from the papers (see Table 3), the same assurance cannot necessarily be granted for wearable sensors, especially considering the evidence that how an eating occasion is defined can significantly influence how eating patterns are characterized^76^. The wide variation in sensor modalities makes this difficult, but also offers us novel research questions that researchers could never consider before (e.g., How is an individual’s number of bites taken throughout the day associated with their risk for developing obesity? Does an individual’s chewing rate predict cardiovascular disease risk? Etc.).

### Future directions

Eating is a highly dynamic behavior that occurs over time and in context. Research has repeatedly shown that contexts have important impacts on eating patterns and health outcomes, including social contexts like eating with family^77–79^, physical contexts like living in a rural food environment^80^, and psychological contexts like chronic stress levels^81,82^. Eating data from wearable sensors, in conjunction with other technologies that can detect these additional contextual features, can be used to eventually develop models of human eating behavior in real time and in context, which can be leveraged to develop adaptive, personalized interventions to predict and change obesity-related behavior, and ultimately, improve population health.

## Conclusions

How do the results from all of these papers inform field work, and how do all of the various sensor modalities reviewed here, really work in the wild? The field is still nascent, and the accuracy of our sensors are still dependent on careful constraints and assumptions. Collecting data in the wild is considerably more challenging than conducting research in controlled settings. In the wild, we lose continuous control of our sensors, participants tamper with the hardware or software, new operating systems bring down our software temporarily. Therefore, successful deployment requires really robust sensing and algorithms that have been tried repeatedly, rather than re-invented again and again. If the goal is to be able to detect eating behaviors in a fully unconstrained manner over a long period of time, novelty can no longer be valued over accuracy. We hope that this review will convince our communities to undertake the hard work and collaboration necessary to develop the kinds of reliable technologies that are required to conduct long-term real-world studies. Developing accurate, robust, automatic and wearable-based eating detection methods will radically reform scientific understanding of eating behavior in time and context and public health.

## Supplementary information

Supplementary Table 1
