# Supplementary material for: Automatic, wearable-based, in-field eating detection approaches for public health research: a scoping review
Source: NPJ Digit Med. 2020 Mar 13;3:38. doi: 10.1038/s41746-020-0246-2 (PMC7069988; doi:10.1038/s41746-020-0246-2)
Supplement: Supplementary file 1 — Supplementary Table 1 [file 41746_2020_246_MOESM1_ESM.pdf]

SUPPLEMENTARY INFORMATION

Supplementary Table 1. Search term strategy

**Database Name**	**Date of Search**	**Search String**	**Result**
ACM Digital Library	12/22/2019	("ambulatory" OR "free living" OR "at home" OR "in field" OR "in the wild") AND (feed% OR eat% OR diet% OR food% OR ingest%) AND ("monitor" OR "assess" OR "detect") AND ("wearable" OR "device" OR "sensor" OR "technology" OR "smart watch" OR "smartwatch")	51
IEEExplorer	12/22/2019	(("ambulatory" OR "free living" OR "at home" OR "in field" OR "in the wild") AND (feed* OR eat* OR diet* OR food OR ingest*) AND ("monitor" OR "assess" OR "detect") AND ("wearable" OR "device" OR "sensor" OR "technology" OR "smart watch" OR "smartwatch"))	95
Google Scholar[1]	12/22/2019	(("ambulatory" OR "free living" OR "in field" OR "in the wild") AND ("eat" OR "food intake" OR "diet") AND ("monitor" OR "assess" OR "detect") AND ("wearable" OR "device" OR "sensor" OR "technology" OR "smart watch" OR "smartwatch"))	50
PubMed	12/22/2019	((Ambulatory OR "free living" OR "at home" OR "in field" OR "in the wild") AND ((((("Nutrition Assessment"[Mesh] AND "Nutrition Surveys"[Mesh] AND "Diet, Food, and Nutrition"[Mesh]) OR "Eating"[Mesh]) OR "Feeding Behavior"[Mesh] OR eating OR "dietary behavior" OR feeding OR "dietary intake" OR "food intake")) AND ((("Monitoring, Ambulatory"[Mesh] OR "ambulatory monitoring")) OR (((Wearable Device OR Electronic Skin Wearable Technology)) OR "Wearable Electronic Devices"[mesh] OR "smart watch" OR "smartwatch" OR "wearable sensor" OR "wearable device")))))	323
Scopus	12/22/2019	(("ambulatory" OR "free living" OR "in field" OR "in the wild") AND ("eat" OR "food intake" OR "diet") AND ("monitor" OR "assess" OR "detect") AND ("wearable" OR "device" OR "sensor" OR "technology" OR "smart watch" OR "smartwatch"))	87

[1]A total of 617 results were produced from this search string, however, only the first 50 results from the Google Scholar database were considered and reviewed.
